# Preliminary Study on Fluidized Bed Chemical Mechanical Polishing (FB-CMP) Process for Stainless Steel 304 (SS304)

**DOI:** 10.3390/mi11070705

**Published:** 2020-07-21

**Authors:** Taekyoung Kim, Hyunseop Lee

**Affiliations:** 1 Department of Mechanical System Engineering, Tongmyong University, Busan 608-711, Korea; wnwkr35@daum.net; 2 School of Mechanical Engineering, Tongmyong University, Busan 608-711, Korea

**Keywords:** fluidized bed finishing (FBM), chemical mechanical polishing, fluidized bed chemical mechanical polishing (FB-CMP), stainless steel

## Abstract

Fluidized bed machining (FBM) is used for the surface finishing or cleaning of complex 3D machine parts. FBM is a process of injecting air into a chamber to encourage particles into a fluid-like state. Subsequently, FBM involves rotating the specimen at high speed to process the surface of the material. However, owing to the long processing time involved in FBM, there is a limit to its application in various industries. In this paper, we propose a fluidized bed chemical mechanical polishing (FB-CMP) process, wherein the material removal mechanism of chemical mechanical polishing (CMP) is applied to FBM to improve the processing efficiency of FBM. An FB-CMP system was prepared, and preliminary experiments on the chemical solution were conducted using stainless steel 304 (SS304) plates. In the experiment, hydrogen peroxide (H_2_O_2_) was used as the oxidant, oxalic acid (C_2_H_2_O_4_) was used as the complexing agent and alumina (Al_2_O_3_) was used as the abrasive particle. The material removal rate (MRR) and roughness reduction rate during the FB-CMP of SS304 were dependent on the composition of the chemical solution. The experimental results revealed the highest MRR and roughness reduction rate at 0.33 wt % H_2_O_2_ and 0.2 wt % oxalic acid. To stabilize the proposed FB-CMP process, it is necessary to examine the chemical solutions of various materials.

## 1. Introduction

Fluidized bed finishing (FBM) has been developed for the surface finishing of three-dimensional parts, and is also termed turbo-finishing [1,2]. FBM is a process of injecting air into a chamber to float abrasives. Subsequently, it involves rotating the specimen at high speed to remove the material, as the abrasive flows in a fluid-like state [1]. FBM is used for edge contouring, deburring, polishing and shot peening in manufacturing parts [3]. The applications of FBM have been expanding, mainly into finishing various types of difficult-to-cut materials and surface cleaning. In general, FBM requires a longer processing time than polishing or grinding in part processing. FBM is known to exhibit a higher processing efficiency than barrel polishing. However, it exhibits a significantly lower material removal rate (MRR) than polishing with a fixed abrasive [3].

Based on Barletta’s study [4], FBM can be used for the surface finishing of aluminum alloys. He applied FBM to the processing of aluminum alloys, and reported on the change in MRR and surface roughness via experiments with alumina (Al_2_O_3_) abrasive. In his experiments, the machining time was up to 40 h and the surface roughness was hundreds of micrometers. Francis et al. [5] examined the swirling abrasive fluidized bed machining of copper with a SiC abrasive. In the study, copper was processed for 14 h, and the amount of material that was removed increased and the surface roughness decreased, then remained constant. Jang et al. [6] investigated the effects of specimen rotation speed and air flow rate on the material removal characteristics of polyacetal in FBM. As the specimen rotation speed increases, the polyacetal MRR increases. However, excessively high air flow lowers the MRR. Overall, FBM requires several hours of processing. Thus, it is necessary to examine techniques for improving the processing efficiency. Recently, Kim and Lee [7] simulated the effects of shear forces on the material via particle motion in the FBM process of material removal, and verified it through experiments. Their study indicated that the shear stress acting on the specimen varies with the rotational speed and air pressure.

Chemical mechanical polishing (CMP) is a process of planarizing the surface of a semiconductor. It is a hybrid material removal process that simultaneously uses chemical reactions and mechanical material removal [8,9,10,11,12]. CMP is a process that mainly processes flat surfaces, such as wafers, and is difficult to apply to the finishing of three-dimensional mechanical structures. In CMP, a slurry containing chemicals and abrasive particles is fed onto a polishing pad to generate a chemically reacted layer on the wafer [13,14,15]. Additionally, the abrasive particles remove the chemically reacted layer on the wafer–pad interface, via the pressure applied to the wafer and the relative movement of the rotating wafer and polishing pad [16,17,18,19].

Stainless steel (SS) is often considered as a material for solar cell substrates; thus, a CMP study on various types of stainless steel was conducted. Hu et al. [20] conducted a CMP study of a flexible stainless foil, using silica particles and hydrogen peroxide (H_2_O_2_) as the oxidant. In their experiments, they showed that a high MRR can be obtained in stainless steel CMP when using a slurry of strong acidic conditions containing H_2_O. In addition to studies conducted by Hu et al., other studies reported a high MRR for acidic slurry [20,21,22]. Chen et al. [23] used alumina slurry for stainless steel CMP, and attempted to determine the optimal slurry composition via a statistical approach. Furthermore, Cheng et al. [24] used alumina slurries and investigated the effects of pressure, rotation speed and particle size on material removal in stainless steel CMP. Jiang et al. [21] conducted a study on stainless steel CMP using a slurry containing colloidal silica, H_2_O_2_, glycine and benzotriazole (BTA). In their studies, acidic slurries exhibited high MRR. Furthermore, Hu et al. [20] observed that oxalic acid (C_2_H_2_O_4_), as a complexing agent, aids in improving MRR. Lee et al. [25] investigated the CMP characteristics of stainless steel 304 (SS304) by using oxalic acid as a complexing agent and H_2_O_2_ as an oxidant. The processing method and mechanism of stainless steel CMP can be applied for improving the processing efficiency of FBM for stainless steel.

The FBM can be applied in the finishing of 3D parts. However, FBM requires a long time for processing. Therefore, it is necessary to increase the processing efficiency of FBM. In this study, we propose a fluidized bed CMP (FB-CMP) process that can improve the processing efficiency of the existing FBM by utilizing the processing mechanisms of FBM and CMP. The proposed FB-CMP process was applied in polishing SS304 plates. Additionally, the existing FBM and proposed FB-CMP were compared and analyzed.

## 2. Fluidized Bed Chemical Mechanical Polishing

**Figure 1:**
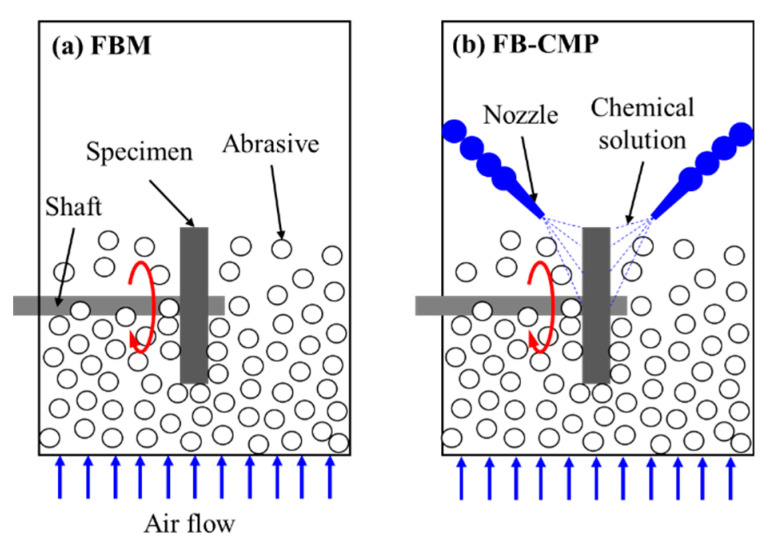
Conceptual diagram for the comparison of fluidized bed machining (FBM) and fluidized bed chemical mechanical polishing (FB-CMP).

**Figure 2:**
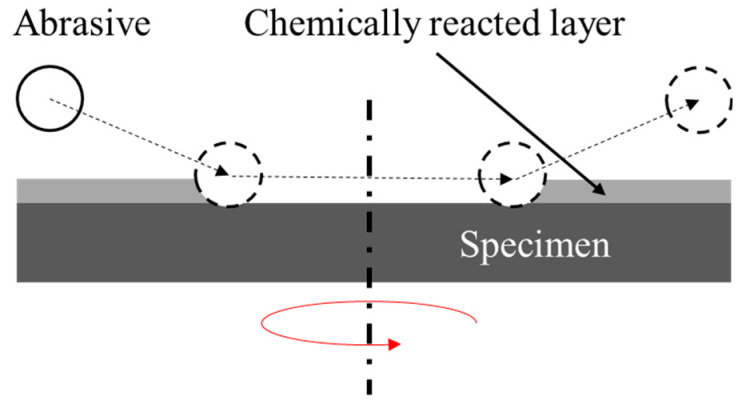
Material removal mechanism of FB-CMP.

FB-CMP was developed to combine the FBM and CMP processing mechanisms for improving the efficiency of existing FBM. In typical FBM, the material is removed mechanically via shear force between the particles floating in the chamber and the rapidly rotating specimen. In a manner similar to CMP, the FB-CMP process exhibits a hybrid processing mechanism that simultaneously utilizes surface chemical reactions and mechanical material removal. In FB-CMP, the chemical solution is sprayed via the nozzles onto both sides of the rotating specimen so as to remove the material via the particles floating in the chamber with air, similar to that in FBM. Figure 1 shows a conceptual diagram that compares FBM and FB-CMP. The material removal mechanism of FB-CMP (Figure 2) is as follows:

1. The surface chemical reaction between the chemical solution and specimen creates a chemically reacted layer that facilitates material removal on the surface of the specimen;
2. The chemically reacted layer on the surface of the specimen is removed by the shear force of the rotating specimen and fluidized abrasive particles;
3. The newly revealed surface of the specimen is repeatedly removed via the process from 1 to 2.

## 3. Experimental

**Figure 3:**
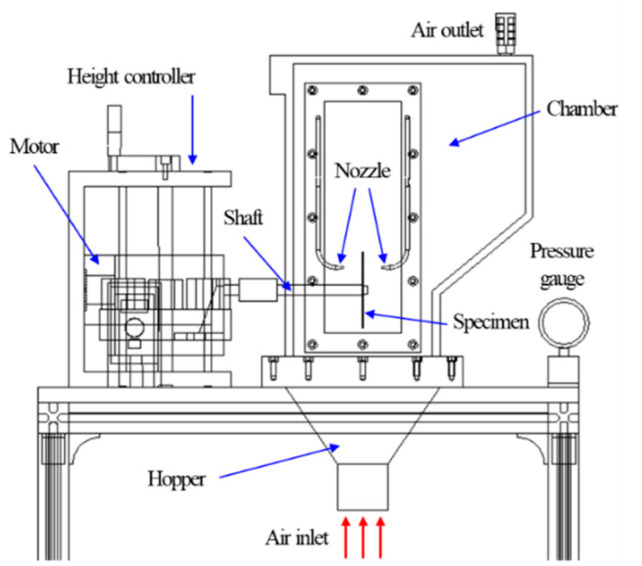
Schematic of fluidized bed chemical mechanical polishing system.

Figure 3 shows the FB-CMP system, which is fabricated for the experiment. The FB-CMP system consists of a chamber, hopper, motor, spindle and height controller. For the abrasives to flow, air is supplied under the chamber and passes through a hopper to float abrasives in the chamber and exit the air outlet. The air passing through the hopper passes through the plenum distributor and is supplied to the chamber. At the end of the shaft, the specimen is mounted, and the shaft is engaged with the motor to rotate the specimen. The height controller controls the height of the specimen in the chamber. During processing, the chemical solution is injected through the nozzles that are located on both sides of the specimen. In this study, a stainless steel (SS304) plate with a diameter of 100 mm and thickness of 0.5 mm was selected as the specimen. Alumina was selected as the abrasive, and the abrasive diameter was 250 μm. The abrasive was filled up to 80 mm from the bottom of the chamber. During the experiment, the abrasive that was used for 1 h was replaced with a fresh abrasive after the experiment.

In the experiment, the air pressure was set to 40 kPa, and the rotation speed of the specimen was 1600 rpm. The processing time was 60 min. The chemical solution for FB-CMP consisted of deionized water (DIW), an oxidizer, and a complexing agent. Furthermore, H_2_O_2_ and oxalic acid were used as oxidizers and complexing agents, respectively. In the experiment, the pH of the chemical solution was fixed at 2.5. The chemical solution was sprayed for 5 s at 10 min intervals to ensure that the abrasive did not adopt a mud-like state due to the solution, and the flow rate was 60 mL/min. Table 1 shows the experimental conditions in detail.

**Figure 4:**
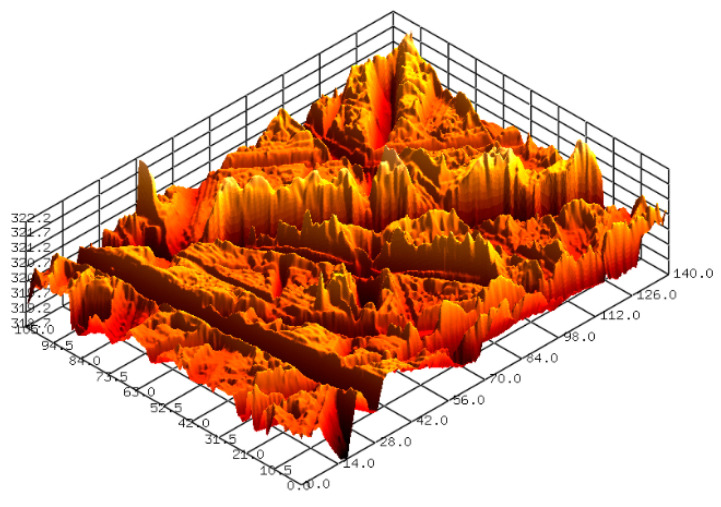
3D surface profile of SS304 after polishing with 80-grit (#80) sandpaper (Sa 0.391 μm).

**Figure 5:**
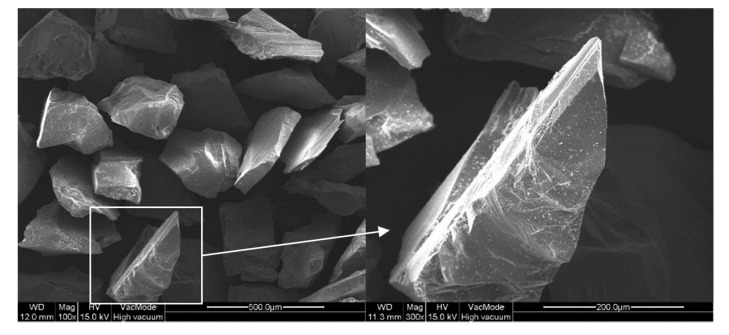
Images of alumina abrasives measured with a scanning electron microscope.

Prior to the FB-CMP experiment, the SS304 plates were polished with 80-grit (#80) sandpapers. The mean surface roughness (Sa) of the SS304 plates was 0.391 μm. Furthermore, the MRR of the specimens was measured using a precision electronic balance before and after FB-CMP. The surface roughness of the specimens was measured using a confocal laser microscope (NS-3500, Nanoscope Systems, Inc., Daejeon, Korea). Surface roughness was measured at four locations on each side of the specimen. The MRR was measured as the change of weight before and after FB-CMP for an hour, and the roughness reduction rate was also calculated as the change of surface roughness before and after FB-CMP for an hour. The Figure 4 shows a representative 3D surface profile of the SS304 plate after polishing with an 80-grit sandpaper. Figure 5 shows the scanning electron microscopy images of the alumina abrasives. The alumina particles used in the experiment have a sufficient cutting edge for processing.

## 4. Results and Discussion

### 4.1. Effect of Hydrogen Peroxide (H_2_O_2_)

Previous studies on stainless steel CMP reported higher a MRR for strongly acidic slurry when H_2_O_2_ is used as the oxidant, compared to that for neutral and basic slurries [20,21,22]. Based on the Pourbaix diagram for Fe, Cr and Ni with water at room temperature [26], the passivation region is generated in the acidic region (pH 2–4). In this study, an acidic chemical solution for FB-CMP was prepared based on previous studies on CMP slurry for stainless steel.

**Figure 6:**
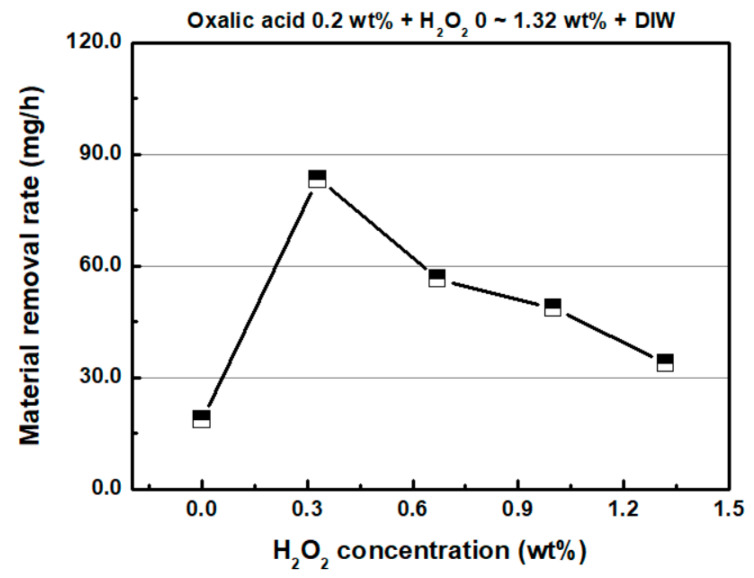
Material removal rate as a function of H_2_O_2_ concentration (oxalic acid concentration: 0.2 wt %).

Furthermore, H_2_O_2_ is widely used as an oxidant in metal CMP. Previous studies also reported the use of H_2_O_2_ as an oxidant in the CMP of stainless steel. The H_2_O_2_ concentration varied from 0 wt % to 1.32 wt %, in order to investigate the effect of oxidizer concentration in the chemical solution on the material removal of SS304 in FB-CMP. Figure 6 shows the MRR as a function of H_2_O_2_ concentration. The concentration of oxalic acid (complexing agent) in the chemical solution was 0.2 wt %. As the H_2_O_2_ concentration increased from 0 wt % to 0.33 wt %, the MRR of SS304 increased from 18.6 mg/h to 83.4 mg/h. When H_2_O_2_ exceeding 0.33 wt % was added, the MRR gradually decreased as the H_2_O_2_ concentration increased. The MRRs at the 0.67, 1.0 and 1.32 wt % H_2_O_2_ concentrations were 56.5, 48.6 and 33.9 mg/h, respectively. The inflection point in the relationship between H_2_O_2_ concentration and MRR in CMP has been reported in other studies [21,22,25,27]. As per the extant studies, excessive H_2_O_2_ in CMP slurry generates a thick passivation layer on the metal surface. The thick passivation layer is difficult to completely remove with abrasives, and this may interfere with the generation of metal ions by the oxidant. This in turn lowers the chemical removal rate via the chelating agent.

**Figure 7:**
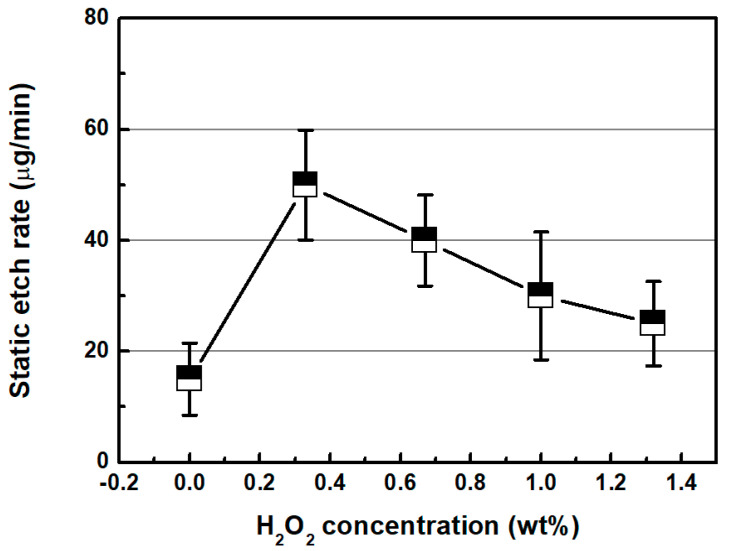
Static etch rate as a function of H_2_O_2_ concentration (oxalic acid concentration: 0.2 wt %).

Figure 7 shows the static etch rate of SS304 based on the hydrogen peroxide content of the chemical solution. The static etch rate was measured in terms of changes in weight after the three SS304 specimens were dipped in a 500-mL chemical solution contained in the beaker for 20 min. Additionally, the static etch rate exhibits the highest value at a hydrogen peroxide concentration of 0.33 wt %. Furthermore, it shows a similar tendency with respect to the changes in MRR, based on the hydrogen peroxide concentration of the chemical solution.

**Figure 8:**
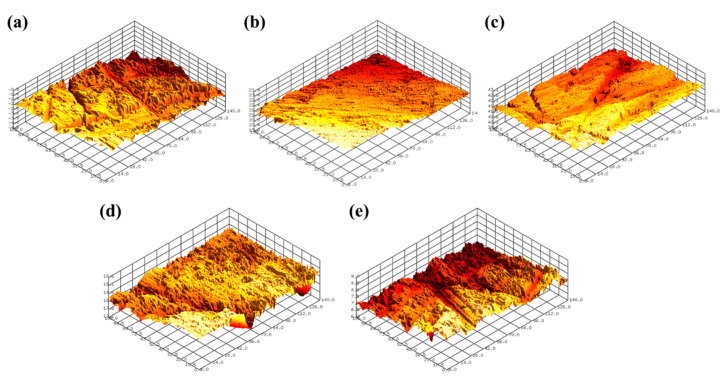
Representative 3D surface profiles of SS304 after FB-CMP with chemical solutions containing various H_2_O_2_ concentrations: (**a**) 0 wt % (Sa 0.219 μm), (**b**) 0.33 wt % (Sa 0.101 μm), (**c**) 0.67 wt % (Sa 0.185 μm), (**d**) 1.0 wt % (Sa 0.213 μm) and (**e**) 1.32 wt % (Sa 0.200 μm).

**Figure 9:**
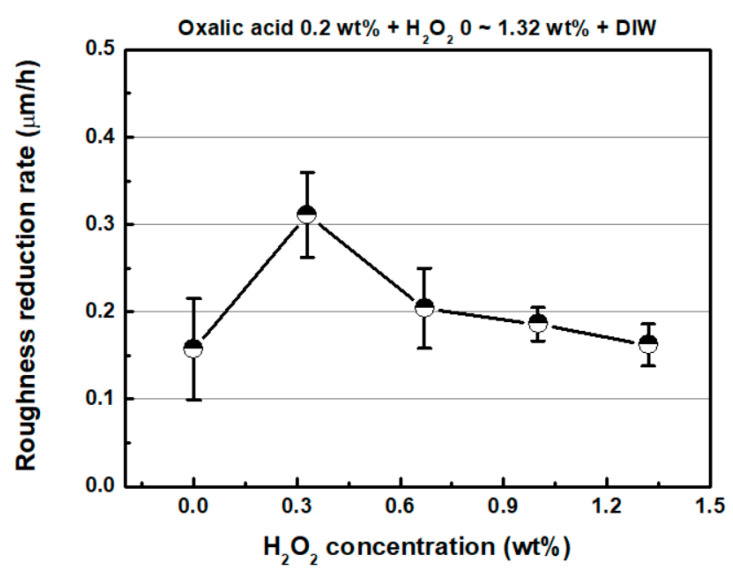
Roughness reduction rate as a function of H_2_O_2_ concentration (oxalic acid concentration: 0.2 wt %).

Figure 8 shows the representative 3D surface profiles of SS304 after FB-CMP with a chemical solution containing various H_2_O_2_ concentrations. Figure 9 shows the roughness reduction rate as a function of H_2_O_2_ concentration. The roughness reduction rate and MRR tended to be similar with respect to the H_2_O_2_ concentration. The roughness reduction rate of SS304 increased from 0.157 μm/h to 0.311 μm/h as the H_2_O_2_ concentration increased from 0.0 wt % to 0.33 wt %. Subsequently, the roughness reduction rate gradually decreased as H_2_O_2_ concentration increased. The roughness reduction rates at the 0.67, 1.0 and 1.32 wt % hydrogen peroxide concentrations were 0.204, 0.186 and 0.162 μm/h, respectively.

### 4.2. Effect of Oxalic Acid

**Figure 10:**
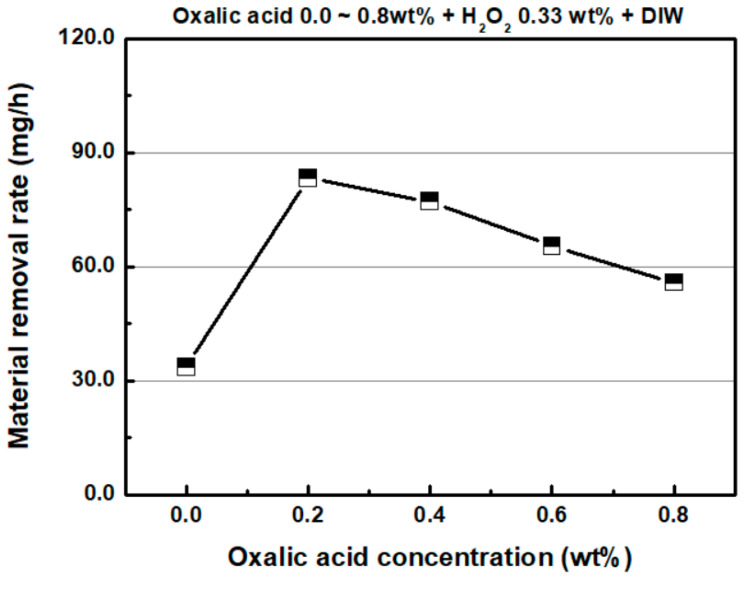
Material removal rate as a function of oxalic acid concentration (H_2_O_2_ concentration: 0.33 wt %).

Previous studies [20,25] reported that the addition of oxalic acid leads to the highest MRR in the CMP of stainless steel when compared to those of the various complexing agents (citric acid, glycine and oxalic acid) used in CMP slurries. The variation in the MRR of SS304 with respect to the oxalic acid concentration in the chemical solution is shown in Figure 10. The H_2_O_2_ concentration in the chemical solution was 0.33 wt %. Without oxalic acid in the chemical solution, the MRR was 33.6 mg/h. When 0.2 wt % of oxalic acid was added to the chemical solution, the MRR increased rapidly to 83.4 mg/h. When more than 0.2 wt % of oxalic acid was added to the chemical solution, the MRR of SS304 decreased continuously. With respect to the oxalic acid concentrations of 0.4, 0.6 and 0.8 wt % in the chemical solutions, the MRRs of SS304 were 77.2, 65.4 and 55.8 mg/h, respectively. In acidic atmospheres, oxalic acid has been sometimes known to act as a corrosion inhibitor for copper or carbon steel [28,29,30]. The decrease in MRR as the oxalic acid concentration exceeded 0.2 wt % may have been due to the aforementioned corrosion inhibition effect.

**Figure 11:**
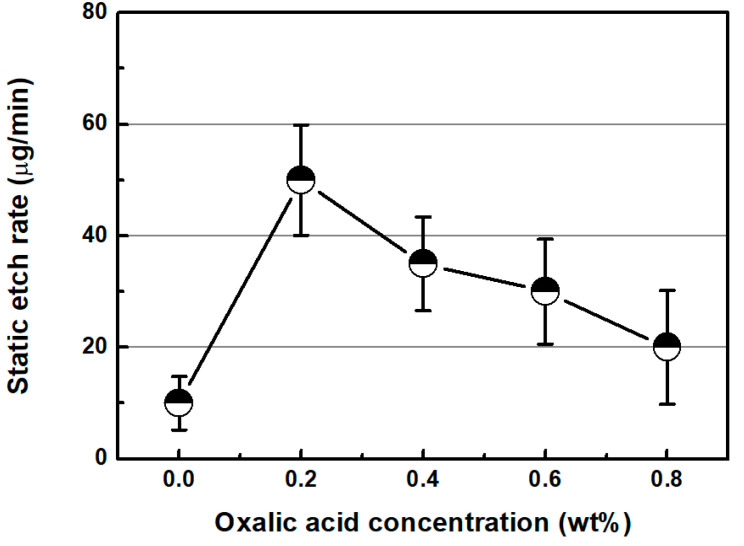
Static etch rate as a function of oxalic acid concentration (H_2_O_2_ concentration: 0.33 wt %).

Figure 11 shows the static etch rate of SS304 with respect to the concentration of oxalic acid. In a manner similar to that of the material removal rate, the static etch rate was the highest at an oxalic acid concentration of 0.2 wt %. Furthermore, as the oxalic acid concentration increased, the static etch rate decreased continuously. The characteristics of static etch rate with respect to the oxalic acid concentration indicate that the change in MRR is due to the chemical reaction between the chemical solution and SS304 specimen.

**Figure 12:**
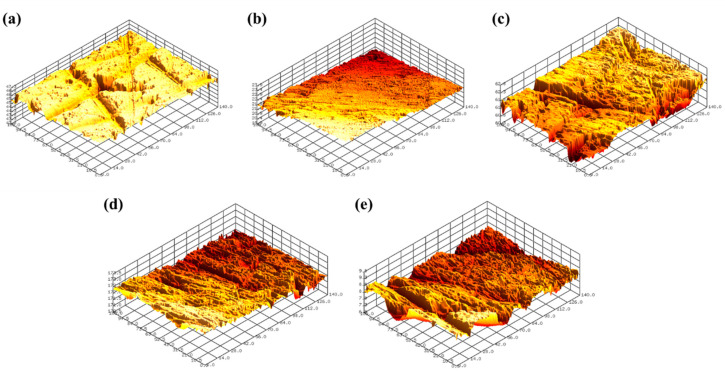
Representative 3D surface profiles of SS304 after FB-CMP for chemical solutions containing various oxalic acid concentrations: (**a**) 0 wt % (Sa 0.280 μm), (**b**) 0.2 wt % (Sa 0.101 μm), (**c**) 0.4 wt % (Sa 0.178 μm), (**d**) 0.6 wt % (Sa 0.180 μm) and (**e**) 0.8 wt % (Sa 0.246 μm).

**Figure 13:**
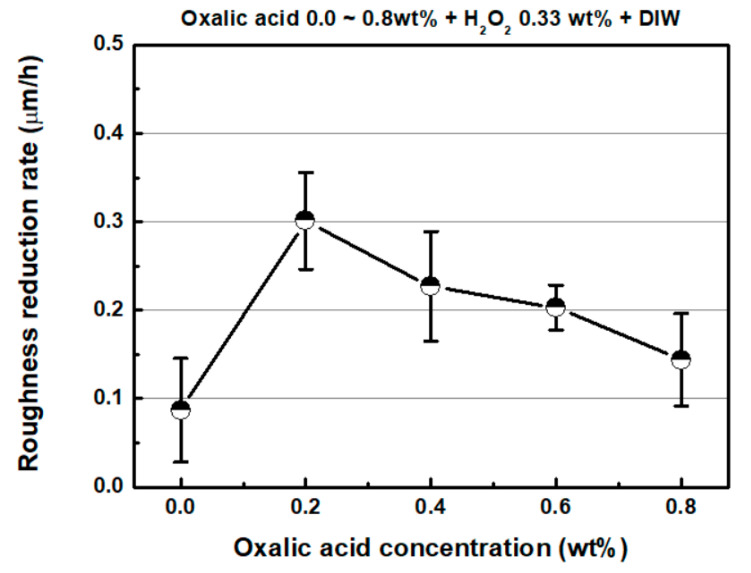
Roughness reduction rate as a function of oxalic acid concentration (H_2_O_2_ concentration: 0.33 wt %).

Figure 12 shows the representative 3D surface profiles of SS304 after FB-CMP for chemical solutions containing various oxalic acid concentrations. Figure 13 shows the roughness reduction rate as a function of oxalic acid concentration in the chemical solution. The roughness reduction rate and MRR tended to be similar with respect to oxalic acid concentration. The roughness reduction rate was 0.111 um/h, when oxalic acid was not added to the chemical solution. When 0.2 wt % oxalic acid was added to the chemical solution, the roughness reduction rate significantly increased to 0.301 µm/h. As the oxalic acid concentration in the chemical solution was increased to 0.4, 0.6 and 0.8 wt %, the roughness reduction rate gradually reduced to 0.227, 0.203 and 0.144 μm/h, respectively.

### 4.3. Comparison of FBM and FB-CMP

**Figure 14:**
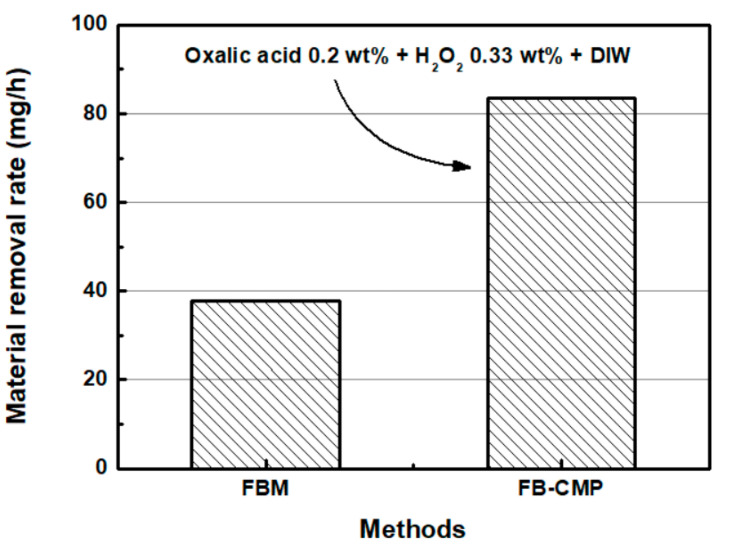
Material removal rates of FBM and FB-CMP (H_2_O_2_ concentration: 0.33 wt %, and oxalic acid concentration: 0.2 wt %).

To compare FB-CMP with conventional FBM, the MRR and roughness reduction rate are shown in Figure 14. In the FBM test, the air pressure was 40 kPa and rotation speed of the specimen was 1600 rpm. Furthermore, 250-μm diameter alumina particles were used as abrasives. The chemical solution for FB-CMP consisted of 0.2 wt % oxalic acid, 0.33 wt % H_2_O_2_ and DIW. The MRR of SS304 after the FBM process was 37.7 mg/h and the MRR SS304 after the FB-CMP process was 83.4 mg/h, under the same air pressure and rotation speed. Specifically, FB-CMP, which simultaneously utilizes the chemical reaction and mechanical material removal, exhibited an MRR approximately 2.2 times greater than that of conventional FBM. The supply of the chemical solution in the FB-CMP appears to increase the efficiency of material removal by creating a chemically reacted layer on the surface of SS304, thereby promoting easy removal of materials.

**Figure 15:**
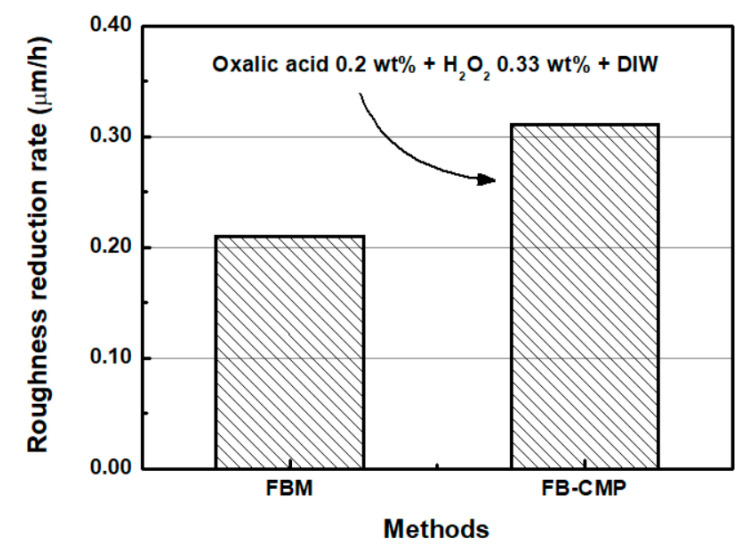
Roughness reduction rates of FBM and FB-CMP (H_2_O_2_ concentration: 0.33 wt %, and oxalic acid concentration: 0.2 wt %).

Figure 15 shows the roughness reduction rates of FBM and FB-CMP. The roughness reduction rates of FBM and FB-CMP were 0.210 μm/h and 0.311 μm/h, respectively. Specifically, a chemical solution containing 0.2 wt % oxalic acid, 0.33 wt % H_2_O_2_ and DIW used in the FB-CMP improved the roughness reduction rate by approximately 1.48 times compared to that of the conventional FBM.

**Figure 16:**
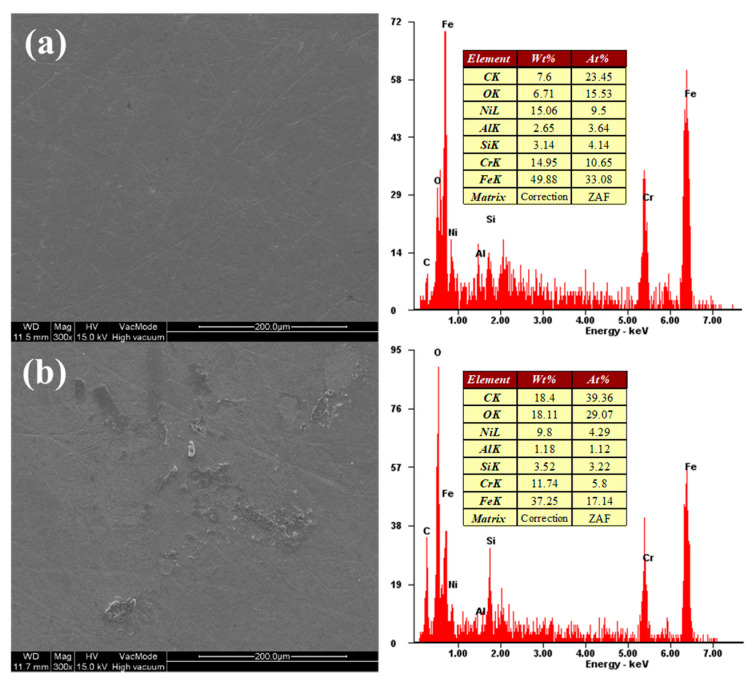
Scanning electron microscope (SEM) images and energy-dispersive X-ray spectroscopy (EDX) analysis results: (**a**) as-received SS304 and (**b**) after dipping in a chemical solution (oxalic acid 0.2 wt %+H_2_O_2_ 0.33 wt %+DIW).

Figure 16 shows scanning electron microscope (SEM) images and energy-dispersive X-ray spectroscopy (EDX) analysis results of SS304 before and after chemical reaction with the chemical solution. The SS304 sample was dipped into the chemical solution for 10 min. The atomic percentage of C and O elements on the surface of SS304, where the chemical reaction occurred due to the chemical solution, was higher than that before the chemical reaction. These results show that in the FB-CMP of SS304, the chemical reaction layer is formed on the surface of SS304 by the oxidant (H_2_O_2_) and complexing agent (oxalic acid) present in the chemical solution.

## 5. Conclusions

In this study, we proposed the FB-CMP, which combines the CMP mechanism and FBM to improve the processing efficiency of the FBM. Specifically, FB-CMP was applied in the processing of a SS304 plate. Furthermore, preliminary experiments on MRR and roughness reduction rate, with respect to the chemical solution composition, were performed. The chemical solution was composed of DIW, H_2_O_2_ as oxidant, and oxalic acid as complexing agent. When the H_2_O_2_ content was changed with an oxalic acid concentration of 0.2 wt %, the MRR and roughness reduction rate were the highest, at 0.33 wt % H_2_O_2_. When the oxalic acid content was changed from 0 wt % to 0.8 wt %, with a constant H_2_O_2_ concentration at 0.33 wt %, the MRR and roughness reduction rate exhibited the highest values at 0.2 wt % oxalic acid concentration. The proposed FB-CMP exhibited higher MRRs and roughness reduction rates than the conventional FBM of SS304. Thus, the FB-CMP can improve the processing efficiency of FBM via a chemical solution. In future studies, research on various process variables, such as solids and fractions associated with air flow rate (or air pressure), the composition of chemicals and the types of particles, will be needed to improve the stabilization and understanding of FB-CMP process. In particular, more detailed studies on the inflection points of MRR and the roughness reduction rate are needed in experiments with changes in the concentration of chemical elements.

## Figures and Tables

**Table 1:** Experimental conditions.

Parameters		Condition
Specimen	Material	Stainless steel (SS304)
	Diameter (mm)	100
	Thickness (mm)	0.5
Abrasive	Material	Alumina (Al_2_O_3_)
	Diameter (μm)	250
Air pressure (kPa)		40
Shaft rotation speed (rpm)		1600
Chemical solution	Base chemical	DIW
	Oxidizer	Hydrogen peroxide (0.0–1.32 wt %)
	Complexing agent	Oxalic acid (0.0–0.8 wt %)
	Flow rate (mL/min)	60
	Injection interval (min)	10 (for 5 s)
Processing time (min)		60
